# Order–Disorder Transitions Govern Kinetic Cooperativity and Allostery of Monomeric Human Glucokinase

**DOI:** 10.1371/journal.pbio.1001452

**Published:** 2012-12-18

**Authors:** Mioara Larion, Roberto Kopke Salinas, Lei Bruschweiler-Li, Brian G. Miller, Rafael Brüschweiler

**Affiliations:** 1 Department of Chemistry and Biochemistry, Florida State University, Tallahassee, Florida, United States of America; 2 Institute of Chemistry, University of São Paulo, São Paulo, Brazil; 3 National High Magnetic Field Laboratory, Florida State University, Tallahassee, Florida, United States of America; Brandeis University, United States of America

## Abstract

Glucokinase (GCK) catalyzes the rate-limiting step of glucose catabolism in the pancreas, where it functions as the body's principal glucose sensor. GCK dysfunction leads to several potentially fatal diseases including maturity–onset diabetes of the young type II (MODY-II) and persistent hypoglycemic hyperinsulinemia of infancy (PHHI). GCK maintains glucose homeostasis by displaying a sigmoidal kinetic response to increasing blood glucose levels. This positive cooperativity is unique because the enzyme functions exclusively as a monomer and possesses only a single glucose binding site. Despite nearly a half century of research, the mechanistic basis for GCK's homotropic allostery remains unresolved. Here we explain GCK cooperativity in terms of large-scale, glucose-mediated disorder–order transitions using 17 isotopically labeled isoleucine methyl groups and three tryptophan side chains as sensitive nuclear magnetic resonance (NMR) probes. We find that the small domain of unliganded GCK is intrinsically disordered and samples a broad conformational ensemble. We also demonstrate that small-molecule diabetes therapeutic agents and hyperinsulinemia-associated GCK mutations share a strikingly similar activation mechanism, characterized by a population shift toward a more narrow, well-ordered ensemble resembling the glucose-bound conformation. Our results support a model in which GCK generates its cooperative kinetic response at low glucose concentrations by using a millisecond disorder–order cycle of the small domain as a “time-delay loop,” which is bypassed at high glucose concentrations, providing a unique mechanism to allosterically regulate the activity of human GCK under physiological conditions.

## Abstract 2

Analysis of the functional dynamics of human glucokinase reveals that a slow order-disorder transition governs monomeric kinetic cooperativity in response to glucose concentrations.

## Author Summary

Glucokinase is a key metabolic enzyme that functions as the body's principal glucose sensor. Glucokinase regulates the rate at which insulin is secreted by the pancreas by using a unique but poorly understood cooperative kinetic response to increasing glucose concentrations. The physiological importance of this enzyme is underlined by the fact that mutations in the glucokinase gene lead to maturity-onset diabetes of the young type II (MODY II), permanent neonatal diabetes mellitus (PNDM), and hypoglycemic hyperinsulinemia of infancy (HI). In this study, we use cutting-edge high-resolution nuclear magnetic resonance methods to understand how the kinetic properties of glucokinase contribute to glucose homeostasis. We also seek to understand how a class of recently discovered small-molecule drugs, which hold promise as therapeutics for type 2 diabetes, function to enhance glucokinase activity. Our results suggest that glucokinase samples a range of conformational states in the absence of glucose. However, in the presence of glucose or a small-molecule activator, the enzyme population shifts towards a more narrow, well-structured ensemble of states. Our findings provide a new model for glucokinase cooperative kinetics, which relies on a slow order–disorder transition in response to glucose concentrations. These results also reveal a universal mechanism of glucokinase activation, which may inform the development of new antidiabetic agents.

## Introduction

Human pancreatic glucokinase (GCK) is the body's principal glucose sensor [1]. GCK is a 52 kDa monomeric enzyme that catalyzes the formation of glucose-6-phosphate from glucose and ATP [2]. This chemical transformation represents the rate-limiting step of glucose catabolism in the pancreas, allowing GCK activity to regulate the rate at which insulin is secreted from β-cells [3]–[5]. The importance of this enzyme in maintaining glucose homeostasis is emphasized by several disease states associated with GCK dysfunction. Loss-of-function mutations, of which more than 600 have been described, cause either maturity onset diabetes of the young type II (MODY-II) or permanent neonatal diabetes mellitus (PNDM) [6]. A small number of gain-of-function mutations have also been identified in patients with the potentially fatal disease, persistent hypoglycemic hyperinsulinemia of infancy (PHHI) [7]–[19]. Functional characterization indicates that most PHHI-associated variant enzymes display a reduced level of positive cooperativity toward glucose. Interestingly, many of the PHHI-associated substitutions co-localize to a common region of the GCK scaffold that is distant from the active site [7]–[19]. In recent years, pharmaceutical research has directed significant efforts toward developing synthetic allosteric activators that target GCK as a strategy to lower blood glucose levels in type 2 diabetic patients [20]. At least one GCK activator has advanced to phase II clinical trials as an antidiabetic agent, yet the functional role and molecular basis of action of such molecules is poorly understood [21]. Similarly, the mechanism of hyperactivity associated with PHHI-linked GCK variants is unclear.

GCK's unique kinetic features are responsible for its physiological role in maintaining glucose homeostasis. The steady-state velocity shows a sigmoidal dependence upon glucose concentration, with a Hill coefficient of 1.7 [2]. This positive cooperativity enables GCK to sensitively respond to small perturbations in blood glucose concentrations, whereby the midpoint of this steady-state response, *K*_0.5_, approximates physiological blood sugar levels (4–10 mM). The kinetic cooperativity of GCK is mechanistically distinct from other modes of allostery, which involve polypeptide oligomerization or require multiple ligand binding sites [22]–[25]. Two theoretical mechanisms—the ligand-induced slow transition (LIST) and mnemonic models—have been put forth to explain kinetic cooperativity in monomeric GCK [26],[27]. In both models, cooperativity is postulated to result from slow conformational transitions that accompany glucose binding and/or product release. The models differ, however, in the relative degree of conformational heterogeneity displayed by the enzyme at various stages along the reaction coordinate. According to the LIST mechanism, two separate catalytic cycles are possible resulting from two distinct conformations of the enzyme. In contrast, the mnemonic model postulates a single catalytically active enzyme conformation with the ability to “remember” its active state for only a short period of time following turnover. Although data have accumulated over the years in support of each mechanism, a consensus has yet to be reached [28]–[33].

**Figure 1:**
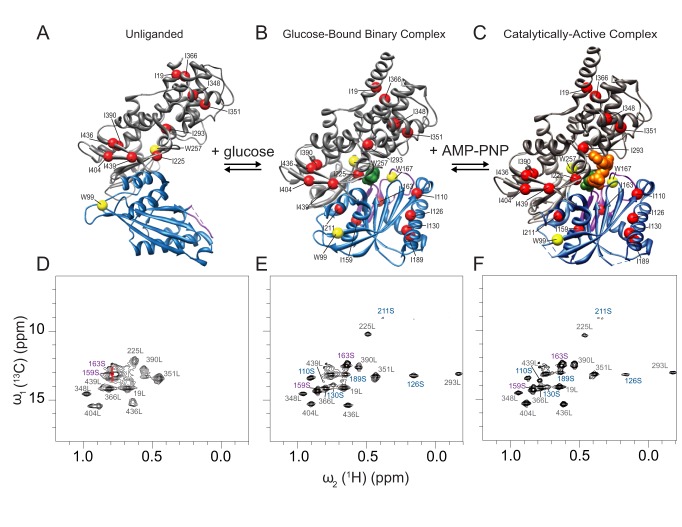
Isoleucine side chain solution NMR spectra along the GCK reaction coordinate. Crystal structures of GCK in (A) unliganded (PDB 1V4T), (B) glucose-bound (PDB 3IDH), and (C) glucose and AMP-PNP-bound forms (PDB 3FGU) depicting the location of isotopically labeled side chains used in this study. Ile Cα positions and tryptophan Cα side chains are depicted as red and yellow spheres, respectively. The large and the small domains are represented in gray and blue, respectively. β-hairpin/loop 151–179 is colored in magenta, glucose is in green, and AMP–PNP in orange. 2D ^1^H-^13^C HMQC NMR spectra of Cδ1 methyl groups of (D) unliganded, (E) glucose-bound, and (F) glucose and AMP–PNP-bound forms of GCK. Assignments of Ile residues in the large domain are in gray with label “L,” Ile residues in the small domain are in blue with label “S,” and Ile residues in the 151–179 loop are in magenta. The average Ile Cδ1 chemical shift of intrinsically disordered Ile side chains deposited in the BMRB database is displayed as an open red circle in Figure 1D (for more details, see Figure S4).

X-ray crystallographic structures reveal that GCK adopts a prototypic hexokinase fold, consisting of a small and large domain separated by a variable opening angle dependent upon substrate association (Figure 1A) [28]. The kinetic mechanism of GCK is strictly ordered [2], with glucose binding first, accompanied by a large structural rearrangement from an open to a closed structure (Figure 1B). Binding of ATP does not induce significant additional changes to the structure of the glucose-bound state (Figure 1C) [34]. The crystal structures also identify a common binding site for synthetic GCK activators at a location within the small domain that is approximately 20 Å removed from the glucose binding site.

Differences observed between available crystal structures demonstrate the ability of GCK to sample discrete conformations, but they do not explain the dynamic basis of allosteric regulation as set forth in the LIST and mnemonic models [28],[29],[34]–[38]. Herein, we describe the results of the first investigation of the functional dynamics of GCK at atomic detail by high-resolution NMR of specifically labeled side chains. We characterize the conformational dynamics of the wild-type enzyme as it progresses along the reaction coordinate and explain the molecular mechanism of kinetic cooperativity. We also uncover the molecular basis of activation observed in PHHI-associated variants or when a synthetic activator associates with the enzyme. Our findings suggest a model for GCK cooperativity involving a slow disorder–order cycle of an intrinsically disordered domain that is operational under low glucose concentrations but that is bypassed at elevated glucose concentrations.

## Results and Discussion

### The Small Domain of GCK Is Intrinsically Disordered

The large size and dynamic nature of GCK, combined with the poor spectral dispersion even upon perdeuteration, prevented the use of traditional NMR approaches for backbone resonance assignment and structural analysis [32]. Instead, we focused on selected side chains only, a strategy that has proven effective for studies of other challenging protein systems and large complexes [39],[40]. For this purpose, we introduced ^13^C spin probes in the Cδ1 methyl groups of 17 isoleucine side chains and ^15^N spin probes at the Nε sites of three tryptophan side chains [39]–[41]. The labeled residues are quite uniformly distributed throughout the enzyme's structure. The large domain contains one Trp and 10 Ile residues, while two Trp and seven Ile residues reside in the small domain (red and yellow spheres in Figure 1A–C). Together these probes permit the study of internal dynamics throughout the protein. Importantly, the methyl resonances can retain narrow line widths even in high-molecular weight proteins through the methyl-TROSY effect manifested in the 2D ^1^H-^13^C HMQC experiment used here [39],[40]. Site-specific assignment of Ile and Trp resonances was achieved by single-site substitutions with Val and Phe, respectively, followed by the recording of 2D ^1^H-^13^C HMQC and ^1^H-^15^N HSQC spectra to identify missing cross-peaks (Figure S1).

To achieve a global portrait of the enzyme's structure and dynamics prior to glucose association, we collected ^1^H-^13^C HMQC spectra of ^13^CH_3_-Ile labeled GCK. The ^1^H-^13^C HMQC of unliganded GCK displays a high degree of cross-peak overlap and heterogeneous peak intensities (Figures 1D and S2). This behavior is consistent with previously reported, unassigned ^1^H-^15^N TROSY spectra of GCK specifically labeled on Ile backbone atoms [32]. Nine Ile residues located in the large domain and two Ile side chains situated in the small domain are observable in unliganded GCK. The 2D ^1^H-^15^N HSQC spectrum of unliganded ^15^N-Trp labeled GCK reveals only a single cross-peak. Mutagenesis demonstrated that this cross-peak is dominated by contributions from W167 (Figure S3). Notably, the only small domain residues observable in the unliganded spectra—I159, I163, and W167—all reside in a loop that is absent in the X-ray structure of unliganded GCK. The sharp cross-peaks originating from these three residues, along with the nearly degenerate chemical shifts of the Ile 159 and 163, reflect the presence of extensive rapid motional averaging and indicate that this loop is disordered, both in the crystal and in solution (Figure S4) [42],[43].

To investigate whether the absence of the resonances of five isoleucines belonging to the small domain results from motions of these side chains occurring on the intermediate exchange regime (µs – ms time scale), we collected ^1^H-^13^C HMQC spectra of unliganded GCK at variable temperatures. Across a range of temperatures (283–313 K) compatible with retention of enzyme activity, no additional cross-peaks were observed that could be attributed to the five missing small domain isoleucine side chains (Figure S5). We tested whether the five missing isoleucine cross-peaks might reside in the intrinsically disordered side chain region of the ^1^H-^13^C HMQC spectrum, underneath the sharp, intense peaks of I159 and I163, by constructing a variant in which I159 and I163 were replaced with leucines. These substitutions had a minimal impact upon the kinetic properties of the enzyme (Table S1); however, the removal of I159 and I163 did not eliminate all peak intensity in the region of the spectrum where disordered side chains are expected. Therefore, residual intensity observed in the I159L–I163L double variant could stem from some of the remaining isoleucine side chains in the small domain (Figure S6). Together these results show that the majority of the isoleucines of the small domain are subject to conformational exchange with an exchange rate *k_ex_* that fulfills the NMR coalescence condition *k_ex_*≈2.2Δ*v*, where Δ*v* is the chemical shift change between conformational substates of unliganded GCK. At least two isoleucines, I159 and I163, remain fully disordered in the unliganded form of GCK.

### Glucose Association Induces Ordering of the Small Domain

**Figure 2:**
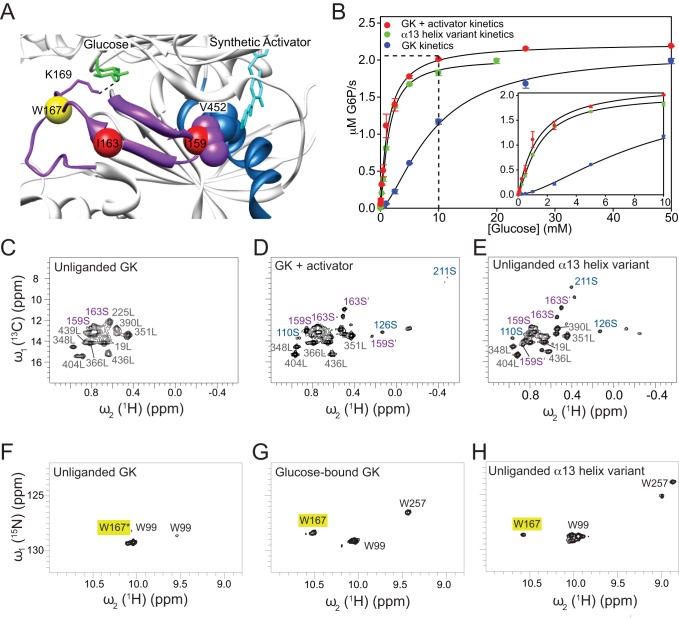
Structural, dynamic, and kinetic descriptions of GCK activating states. (A) Crystal structure of the GCK–activator complex depicting the spatial vicinity of the regions involved in allosteric communication. The 151–179 β-hairpin is shown in magenta, the α13-helix in blue, and the Cαs of Ile-159, Ile-163, and Trp-167 residues are colored in red (Ile) and yellow (Trp) spheres. Glucose is colored in green, and the synthetic allosteric activator in cyan. The side chain of Lys-169, which is represented by magenta sticks, is hydrogen-bonded to O6 of glucose. Val-452 and Ile-159 side chains are located within 5 Å of each other and of the activator thiazole ring. (B) Kinetic response of the α13-helix variant (green), and wild-type GCK in the absence (blue) and presence (red) of saturating activator (see Table S1). 2D ^1^H-^13^C HMQC spectra of (C) unliganded GCK, (D) activator-bound complex, and (E) unliganded α13-helix variant, specifically labeled with Ile ^13^C(δ1) methyl groups. 2D ^1^H-^15^N HSQC spectra of (F) unliganded GCK, (G) glucose-bound complex, and (H) unliganded α13-helix variant, with Trp-^15^Nε cross-peaks assigned by black labels. The yellow box highlights the position of the W167 cross-peak. The asterisk indicates that W167 is the main contributor to the intensity of the unliganded cross-peak (for more details, see Figure S3).

Upon addition of glucose, the intensity of the intrinsically disordered region of the ^1^H-^13^C HMQC spectrum decreases and new cross-peaks originating from I110, I126, I130, I189, and I211 become clearly visible (blue peaks in Figure 1E). In contrast, residues in the large domain are much less affected by glucose binding (gray peaks). In the presence of glucose, the cross-peaks of I159 and I163 dramatically increase their line widths and shift to new positions (Figures 1E and S2), reflecting the formation of the 151–179 β-hairpin, a well-defined structural element in the crystal structure 1V4S [28]. In the ^1^H-^15^N HSQC spectrum of the ^15^N-tryptophan-labeled GCK–glucose complex, three well-resolved cross-peaks corresponding to W99, W167, and W257 are observed (Figure 2G), consistent with the glucose-induced structural organization observed in the ^1^H-^13^C HMQC spectra of ^13^CH_3_-Ile labeled enzyme. Addition of the ATP analogue AMP–PNP to the binary complex perturbs the spectrum only slightly, indicating that AMP–PNP weakly impacts the structure and dynamics of the binary complex (Figure 1F).

### Molecular Basis of Activation for PHHI-Associated Substitutions and Therapeutic Agents

Although PHHI is usually associated with single amino acid replacements, we employed our previously identified hyperactive quadruple variant to fully characterize the structural and functional impacts of activating mutations [44]. This hyperactive variant contains a redesigned α13-helix with sequence ALIAAAV. Similar to other activating PHHI-linked variants, the α13-helix variant displays a decreased glucose *K*_0.5_ value, a reduced level of cooperativity, and an increased equilibrium affinity for glucose compared to wild-type GCK [44]. Specifically, the glucose *K*_D_ value of the α13-helix variant is 50 µM, a value similar to glucose *K*_m_ values of the noncooperative GCK isozymes, hexokinases I–III. In contrast to wild-type GCK, the ^1^H-^13^C HMQC of the unliganded α13-helix variant displays cross-peaks originating from Ile residues in both the small and large domains (Figure 2E). Under saturating glucose conditions, the α13-helix variant exhibits a spectrum that closely resembles the wild-type glucose-bound spectrum (Figure S7). These spectral characteristics are not specific to the hyperactive α13 variant, as similar effects were observed with the single-site activating variant, S64P (Figure S8B). In general, the ^1^H-^13^C HMQC spectra of activated variants reveal varying degrees of structural stabilization relative to the wild-type enzyme. We observed a correlation between increases in glucose affinity caused by individual activating GCK variants, a systematic sharpening of cross-peaks, and the appearance of a larger number of resolved cross-peaks in the ^1^H-^13^C HMQC spectra in their unliganded state (Figure S8). These data demonstrate that activating PHHI-associated substitutions alter enzyme dynamics and promote a conformational ensemble that more closely resembles the glucose-bound state.

Binding of the allosteric activator, which reduces the glucose *K*_0.5_ value and eliminates cooperativity, induces changes in the 2D ^1^H-^13^C HMQC spectrum similar to that produced by activating variants (Figure 2). Well-dispersed cross-peaks originating from Ile residues in both the large and small domain are observed in the presence of the activator. The only differences between the NMR spectra of glucose-bound GCK in the absence and presence of the activator arise from residues near the activator binding site, such as I211 (Figure S9), suggesting local perturbations only. From these data, we conclude that allosteric activators stabilize the small domain and alter GCK dynamics. Our results also demonstrate that small-molecule-mediated GCK activation does not require prior formation of the binary GCK–glucose complex, a finding that could have important therapeutic implications.

### Direct Mode of Communication between the Allosteric and Active Sites: Stabilization of the α13-Helix Leads to Folding of the 151–179 Loop

The 151–179 loop is disordered in unliganded wild-type GCK, as evidenced by the fact that the chemical shifts and peak intensities of I159, I163, and W167 fall within the disordered side chain region (Figure S4). This loop becomes structured in the unliganded α13-helix variant as shown by the 2D ^1^H-^15^N HSQC and 2D ^1^H-^13^C HMQC spectra in Figure 2E and H. Notably, the chemical shift of W167 in the unliganded α13-helix variant is identical to that observed in the wild-type GCK-glucose complex (Figure 2G and H), indicating that the loop conformation adopted by the variant is similar to that produced upon glucose binding. The crystal structure reveals a possible mode of communication between the activator, the α13-helix, and the 151–179 loop (Figure 2A) [28]. One face of the activator binding site is comprised of the α13-helix residues V452, V455, and A456, and two of these residues, V452 and A456, interact with the loop residue I159. In the presence of the activator, or when an activating substitution is introduced into the α13-helix, the interactions between I159, V452, and A456 are stabilized. In turn, this stabilization is relayed to the glucose binding site via two additional loop residues, T168 and K169, which form hydrogen bonds with the O1 and O6 atoms of bound glucose. Structural organization of either the α13-helix or the 151–179 loop can be achieved by glucose binding, activator association, or a hyperactive substitution.

### Our Data Reveal a Universal Mechanism of GCK Activation

PHHI variants or the allosteric activator promote a population shift toward a more structured state, which involves a major reorganization of the small domain. This effect, which is evidenced by increased spectral dispersion and loss of line broadening compared to the unliganded wild-type enzyme, abrogates kinetic cooperativity. The differential glucose binding affinities of wild-type GCK and the α13-helix variant (*K*_D_ = 5 mM and 50 µM, respectively) suggest that a substantial thermodynamic barrier separates the closed GCK conformation from the unliganded state. In the wild-type enzyme, this barrier is overcome by glucose binding, which triggers the disorder–order transition. In an activated variant, or in the presence of a synthetic activator, the small domain is stabilized, which decreases the free energy penalty of the conformational change and allows glucose binding energy to manifest itself in the form of a lower *K*_D_ value. The differential glucose binding affinities of wild-type and activated GCK also suggest that the high-affinity conformation represents ≤1% of the total unliganded ensemble.

### A Refined Model for GCK Cooperativity

The experimental data presented here provide direct evidence that the small domain of GCK is highly dynamic in the absence of ligand. Some evidence of GCK structural plasticity has emerged from X-ray structures determined in the presence and absence of various ligands; however, since crystal structures represent high-resolution snapshots of protein states, they neither reveal time-scale information nor how different states are dynamically related to each other. Optical spectroscopy, on the other hand, provides time-scale information at single sites without reporting on local structure. Indeed, the application of transient state fluorescence spectroscopy to GCK revealed the existence of glucose-induced structural rearrangements that span multiple time scales, but these studies failed to provide a unified, structure-based mechanism of GCK cooperativity [45]–[48]. Here we used all native Ile and Trp side chains to probe the structural-dynamic behavior of GCK at 20 different sites distributed throughout the protein. These data, obtained in solution under physiological conditions in the absence and presence of glucose and activator, provide temporal information for wild-type GCK and its variants with high spatial resolution. Based on this new type of information, we propose a refined model for allostery in monomeric GCK.

**Figure 3:**
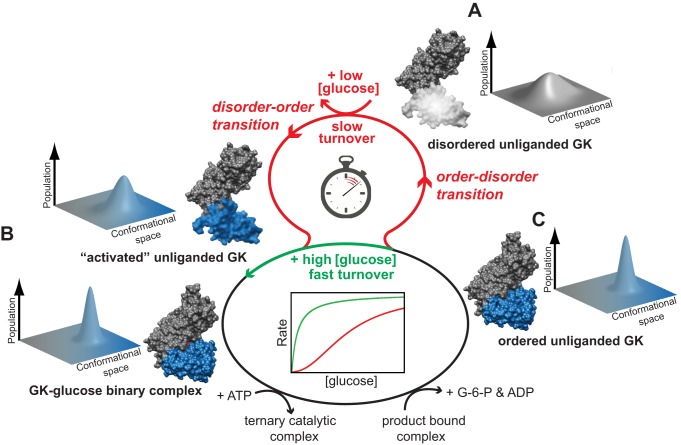
Mechanism of GCK kinetic cooperativity. (A) The small domain of unliganded GCK is intrinsically disordered, giving rise to a broad conformational ensemble. (B) Glucose binding, activator binding, or an activating PHHI-associated mutation promotes folding of the disordered regions in the small domain, narrowing the conformational distribution. Upon formation of the GCK–glucose binary complex, ATP binds and catalysis proceeds with little additional reorganization. (C) Following product release, ordered unliganded GCK persists until the small domain undergoes an order–disorder transition on the millisecond time scale, allowing access to the “time delay loop” (red): Under low glucose concentrations, the delay loop is operational, leading to slow turnover and kinetic cooperativity. Under high glucose concentrations (or when GCK is activated), the delay loop is effectively bypassed, turnover is fast, and cooperativity is eliminated (green).

The model that emerges from our data demonstrates that GCK cooperativity may result from dynamic structural modulations of the intrinsically disordered small domain. In the absence of ligand, the enzyme exists as an ensemble of conformations, which interconvert on a millisecond time scale that coincides with enzyme turnover (*k*_cat_∼60 s^−1^) (Figure 3). Upon glucose association the small domain becomes ordered, as reflected in the sharpening of the NMR resonances and the increase of chemical shift dispersion of Ile residues. After formation of the GCK–glucose binary complex, ATP binds and catalysis proceeds with little additional reorganization. Following product release, ordered unliganded GCK persists until, on the millisecond time scale, the small domain undergoes an order–disorder transition, allowing access to a “time delay loop.” Under low glucose concentrations, the delay loop is operational, leading to slow turnover and kinetic cooperativity (Figure 3, red). Under high glucose concentrations, or when GCK is activated, the delay loop is effectively bypassed, turnover is fast, and cooperativity is eliminated (Figure 3, green).

The putative involvement of slow conformational changes in the generation of kinetic cooperativity in enzymes has long been appreciated [49]. In the case of GCK, the generation of a sigmoidal steady-state response, which is key to this enzyme's sensitivity to oscillating physiological glucose levels, requires a time delay slower than 1/*k*_cat_. The absence of resonances from Ile residues located in the small domain prevents quantitative analysis of the underlying rate constants associated with the disorder–order transition. However, typical Ile and Trp side-chain chemical shift ranges point to a rate constant *k_ex_* between 5 s^−1^ and 100 s^−1^ in order to explain the disappearance of the cross-peaks from the small domain by coalescence. This disorder–order transition rate satisfies the conditions needed to account for the existence of kinetic cooperativity in GCK [26],[27]. Protein structural organization occurring in the millisecond time regime is not uncommon [50], and here this phenomenon appears to contribute to the generation of GCK cooperativity. As with other intrinsically disordered proteins [51],[52], it is possible that the dynamic nature of the small domain also plays a role for the emergence of new functional attributes, including regulation of GCK activity by multiple interacting partners [53]–[55] and posttranslational events [56],[57].

## Materials and Methods

### Protein Expression and Purification

Recombinant human pancreatic GCK was produced as an N-terminal hexa-histidine-tagged polypeptide in *Escherichia coli* strain BL21(DE3). Bacterial cultures were inoculated to an initial OD_600 nm_ of 0.06 and were grown at 37°C in minimal medium supplemented with ampicillin (150 µg/mL), thiamine hydrochloride (25 µg/mL), ^15^NH_4_Cl (1 g/L), Ca(OH)_2_ (0.1 mM), MgSO_4_ (1 mM), and glycerol (1%) (w/v). When the OD_600 nm_ reached 0.85, IPTG (1 mM) was added to induce gene expression and growth was continued for 12 h. The specific incorporation of ^15^N and ^13^C labels in the Trp and Ile residues, respectively, was achieved following the protocols by Muchmore et al. [41] and Tugarinov et al. [39]. Cells were harvested by centrifugation at 8,000 *g*, and 5 g of wet cell pellet was resuspended in 17 mL of buffer A containing HEPES (50 mM, pH 7.6), KCl (50 mM), imidazole (40 mM), dithiothreitol (10 mM), and glycerol (25% w/v). Cells were lysed using a French Press and subjected to centrifugation at 25,000 *g* at 4°C for 1 h. The supernatant was immediately loaded onto a 5 ml HisTrap Fast Flow Affinity Column (GE Healthcare) previously equilibrated in buffer A. Following loading, the column was washed with 10 column volumes of buffer A followed by 5 columns of buffer A containing 65 mM imidazole. GCK was eluted with buffer A containing 250 mM imidazole, and the enzyme was dialyzed at 4°C against 1L of potassium phosphate buffer (25 mM, pH 8), containing KCl (25 mM) and dithiothreitol (10 mM). GCK was then concentrated to ∼600 µM using an Amicon centrifugal concentrator (MWCO = 10,000). Protein was injected onto a Superdex 200 16/60 gel filtration column (Amersham-Pharmacia) pre-equilibrated with potassium phosphate buffer (25 mM, pH 8.0), containing KCl (25 mM) and DTT (10 mM). The gel filtration column was run at a flow rate of 0.12 mL/min, and fractions that contained the highest A_280 nm_ readings were pooled and used immediately in the NMR experiments.

### Site-Directed Mutagenesis

Site-directed mutagenesis was performed using the QuikChange protocol (Stratagene). Mutagenesis reactions contained human *glk* template DNA (400 ng), Pfu Turbo enzyme (2.5 units), cloned Pfu Turbo reaction buffer (1×), and mutagenic primers (125 ng).

### Enzyme Assays

GCK activity was measured spectrophotometrically at 340 nm by coupling the production of ADP to the oxidation of NADH via the combined action of pyruvate kinase and lactate dehydrogenase. Assays were conducted at 25°C in reaction mixtures containing HEPES (250 mM, pH 7.6), KCl (50 mM), NADH (0.25 mM), dithiothreitol (10 mM), pyruvate kinase (15 units), lactate dehydrogenase (15 units), ATP (0.1–50 mM), MgCl_2_ (1.1–51 mM), and glucose (0.05–100 mM). Data were fitted to the Hill equation or the Michaelis-Menten equation depending on the substrate under investigation. Assays were initiated by the addition of ATP and were conducted in duplicate for each substrate concentration. The kinetic constants reported are the average of data obtained from at least two independent preparations of enzyme. Assays were conducted before and after NMR measurements to verify retention of enzyme activity during the time course of the experiment.

### NMR Data Collection, Processing, and Resonance Assignment

All NMR data were collected with a Bruker Avance III spectrometer operating at 800 MHz proton field and equipped with a TCI cryogenic probe. The GCK NMR samples were prepared in potassium phosphate buffer (25 mM, pH 8.0), containing KCl (25 mM), DTT (10 mM), deuterated glycerol (5% v/v), and D_2_O (10% v/v). For all experiments of glucose-bound GCK, glucose was added to a total concentration of 200 mM. ^1^H-^13^C HMQC experiments were recorded as matrices of 2048×390 (in Figure 1) or 2048×256 (in Figure 2) complex data points. Unless otherwise stated, ^1^H-^15^N HSQC experiments were recorded as matrices of 2048×128 complex data points. All spectra were apodized with a cosine function in each dimension prior to zero-filling. NMR data processing and spectral analyses were performed with NMRPipe [58] and the CCPN-Analysis software [59]. As a control, the enzymatic activity of GCK was measured before and after each NMR experiment.

Site-specific assignments of Ile and Trp resonances were mainly achieved by single-site substitution with Val/Leu and Phe, respectively, followed by the recording of 2D ^1^H-^13^C HMQC and ^1^H-^15^N HSQC spectra to identify missing cross-peaks (Figure S7). Assignments of Ile residues in the α13-helix variant could be directly transferred from their wild-type assignments due to identical peak positions, except for I159 and I163, which were assigned by site-directed mutagenesis. Ile residues in the GCK–activator complex were assigned based on identical cross-peak positions with respect to the wild-type spectrum. Assignments for W99, W167, and W257 in wild-type GCK and for W167 in the α13-helix variant were performed by individually replacing Trp by Phe.

## Supporting Information

Figure S1 Illustration of the assignment procedure for Ile cross-peaks of GCK. The spectrum of wild-type GCK in the glucose-bound state (black) is superimposed on the spectrum of the I351V variant (red). The disappearance of the cross-peak due to I351 in the mutant is followed by a small chemical shift change of I348 due to their close spatial proximity.
(EPS)

Figure S2 Distribution of NMR cross-peak line widths and intensities of GCK in the absence and presence of glucose. The 2D ^1^H-^13^C HMQC cross-peaks of unliganded GCK display a broad range of line widths along the ^13^C dimension between 10 and 115 Hz. (A) Addition of glucose narrows the line width distribution to a range of 20–60 Hz. (B) Unliganded GCK shows a heterogeneous distribution of ^1^H-^13^C HMQC cross-peak intensities, which becomes more homogeneous upon glucose addition. Labels indicate the intensities and line widths of I159 and I163 signals as they are the strongest and sharpest cross-peaks in the absence of glucose, which is consistent with their high internal mobility. The ^1^H-^13^C HMQC experiments were recorded with t_1_-evolution times up to 56.59 ms. The spectra were zero-filled to 8,192 data points in both dimensions and processed without apodization.
(EPS)

Figure S3 Trp167 is the major contributor to the dominant cross-peak in the ^1^H-^15^N HSQC spectrum of unliganded GCK. One-dimensional cross-sections along the ω_2_ (^1^H) dimension corresponding to a ω_1_ (^15^N) frequency of 129 ppm, extracted from 2D ^1^H-^15^N HSQC spectra of wild-type GCK (black), W99F (blue), W257F (green), and W167F variant (red). All ^1^H-^15^N HSQC spectra were recorded as matrices of 2048×68 complex data points and zero-filled to 2048×128 data points. The NMR samples consisted of 370, 330, 532, and 257 µM protein solution for W167F, W99F, W257F, and wild-type GCK, respectively, labeled specifically with ^15^N at all tryptophan side chains. The spectra were uniformly scaled by factors of 1.12 (W99F), 2.04 (wild-type), and 0.69 (W257F) in order to account for the different sample concentrations relative to the W167F sample.
(EPS)

Figure S4 Ile and Trp side-chain chemical shifts reflect the disordered nature of unliganded GCK. (A) The ^13^C and the ^1^H chemical shifts of I159 and I163 (Cδ1 and Hδ1) observed in the ^1^H-^13^C HMQC spectrum of unliganded GCK are consistent with a disordered loop, as evidenced by comparison with the ^13^C and ^1^H chemical shifts of Ile ^13^Cδ1-^1^Hδ1 spin pairs of denatured or intrinsically disordered proteins deposited in the Biological Magnetic Resonance Data Bank or BMRB (solid red circles). The mean of the BMRB distribution of disordered Ile ^13^Cδ1-^1^Hδ1 is shown as a solid green circle, with the error bars corresponding to the standard deviations along the ^13^C and ^1^H dimensions: <δ(^13^C)> = 12.689±0.449 ppm and <δ(^1^H)> = 0.785±0.054 ppm. The dark blue circle represents an outlier, which is >11 standard deviations away from the average Ile ^13^Cδ1 chemical shift belonging to BMRB entry 7279 and which was excluded from analysis. The small blue dots represent Ile ^13^Cδ1-^1^Hδ1 chemical shifts of all BMRB entries, and their average is indicated by the cyan open circle. (B) The ^15^Nε1 and the ^1^Hε1 chemical shifts of the Trp167 side chain observed in the ^1^H-^15^N HSQC spectrum of unliganded GCK are also consistent with a disordered region. The solid green circle shows the mean of the BMRB distribution of disordered Trp ^15^Nε1-^1^Hε1 along the ^15^N and ^1^H dimensions: <δ(^15^N)> = 129.459±0.118 ppm and <δ(^1^H)> = 10.087±0.016 ppm. The dark blue circle represents an outlier, which is >38 standard deviations away from the average Trp ^1^Hε1 chemical shift belonging to BMRB entry 15201 and which was excluded from analysis. The small blue dots represent Trp ^15^Nε1-^1^Hε1 chemical shifts of all BMRB entries, and their average is indicated by the cyan open circle. The red solid circles represent Trp ^15^Nε1-^1^Hε1 chemical shifts of denatured or intrinsically disordered proteins deposited in the Biological Magnetic Resonance Data Bank or BMRB. (A and B) Chemical shifts for denatured or intrinsically disordered proteins were obtained from a subset of all BMRB entries marked as “unfolded.” The analysis was restricted to those entries that (i) have assigned side-chain Ile ^1^Hδ1-^13^Cδ1 and Trp ^15^Nε1-^1^Hε1 chemical shifts and (ii) have local secondary structure propensity scores (SSP) at the isoleucine and tryptophan sites between −0.3 and 0.3 [60]. The second criterion, which ensures that protein regions with relatively high (>30%) propensities for α13-helix or β-strand propensities are excluded from analysis, was calculated as the SSP averaged over residues (i−1, i, i+1), where i is Trp or Ile. This left three BMRB entries (15201, 15225, and 7279) for Ile ^1^Hδ1-^13^Cδ1 and two entries (15201 and 15225) for Trp ^15^Nε1-^1^Hε1.
(EPS)

Figure S5 The influence of temperature on the 2D ^1^H-^13^C HMQC of unliganded GCK. Comparison of high-temperature (313K, A) with low-temperature (283K, B) ^1^H-^13^C HMQC of unliganded GCK. A decrease in signal intensity and disappearance of cross-peaks is observed upon decreasing temperature by 30°C.
(EPS)

Figure S6 Residual intensity exists underneath the sharp peaks of I159 and I163 and displays glucose dependence. Superposition of the first increment of 450 µM I159LI163L (red) 2D ^1^H-^13^C HMQC with the 400 µM wild-type GCK (blue) in the unliganded (A) and glucose-bound form (B). In unliganded GCK (A, blue spectrum) the intensity of the intrinsically disordered side-chain region is dominated by I159 and I163. Substitution of these isoleucine residues with leucine revealed the existence of residual intensity in this region attributable to some or all of the other isoleucine residues located in the small domain (A, red, spectrum). Addition of saturating glucose to the variant lacking reporters in the disordered loop region (I159LI163LGCK, B, red spectrum) decreased further the intensity of the intrinsically side chain region compared to the wild-type GCK (B, blue spectrum). The data were recorded as matrices of 1024×64 (wild-type) and 1024×350 (I159LI163L GCK) complex data points and multiplied by a squared cosine apodization function.
(EPS)

Figure S7 The hyperactive α13-helix variant of GCK adopts a virtually identical conformation as wild-type GCK upon glucose binding. (A) Overlay of the 2D ^1^H-^13^C HMQC spectrum of glucose-bound α13-helix variant (red) and wild-type GCK (black) upon addition of 200 mM glucose. The only significant difference in the spectra concerns I211, which is present as a very weak cross-peak in wild-type GCK. (B) Superposition of 2D ^1^H-^13^C HMQC spectrum of unliganded α13-helix variant (red) and glucose-bound GCK (black). Note that both I163 and I159 appear as peak pairs, with one of the peaks each coinciding with the peak positions of unliganded wild-type GCK. The other two peaks of I159 and I163, marked with a prime, appear at positions that are further away from the disordered region, indicating a certain degree of ordering.
(EPS)

Figure S8 Correlation between glucose binding affinity, cross-peak dispersion, and line widths for different activated states of GCK. (A) The 2D ^1^H-^13^C HMQC spectrum of unliganded GCK displays significant spectral overlap and broad cross-peaks. (B and C) Activating states of GCK show increased affinities for glucose binding and display a gradual increase in cross-peak dispersion and peak narrowing. Wild-type GCK has a *K*_D, glucose_ of 5 mM, S64P variant has an intermediate *K*_D, glucose_ of 120 µM, and the α13-helix variant displays a *K*_D, glucose_ of 50 µM.
(EPS)

Figure S9 NMR characterization of activator binding to the binary GCK-glucose complex and glucose titration to GCK. (A). Activator binding to the binary GCK–glucose complex induces only local chemical shift changes without perturbing the conformation of the binary state. 2D ^1^H-^13^C HMQC of GCK–glucose binary complex in the absence (black) and presence (red) of saturating activator. An arrow indicates a significant chemical shift change induced by activator binding. The most affected residues (I159, I404, and I211) are labeled in red and are located in close proximity to the allosteric binding site. I211 interacts directly with the activator. I159 is located within 5.8 Å from the N of the thiazole ring of the activator, and I404 is located within 9 Å from the methyl carbon of imidazole ring of the activator molecule. (B) 2D ^1^H-^13^C HMQC of GCK in the absence of glucose (left) and displaying the cross-peak of I436 (right) for clarity. (C) 2D ^1^H-^13^C HMQC of GCK in the presence of physiologically relevant glucose concentrations (5 mM) (left) where the cross-peak of I436 (right) displays two populations corresponding to the unbound and glucose-bound fractions, respectively. (D) 2D ^1^H-^13^C HMQC of GCK in the presence of saturating amounts of glucose (left) where the I436 cross-peak (right) is represented just by the glucose-bound population. The results of (B, C, D) reveal that the unliganded and glucose-bound states of GCK are in slow exchange on the NMR time scale.
(EPS)

Table S1 ^a^Kinetic parameters of wild-type and variants of GCK used in this study.
(DOCX)

## Abbreviations

^1^H-^13^C HMQC: Heteronuclear Multiple Quantum Coherence
^1^H-^15^N HSQC: Heteronuclear Single Quantum Coherence
ATP: adenosine-5′-triphosphate
DTT: dithiothreitol
GCK: glucokinase
HEPES: 4-[2-hydroxyethyl]-1-piperazineethanesulfonic acid
IPTG: Isopropyl β-D-1-thiogalactopyranoside
LIST: ligand-induced slow transition
MODY-II: maturity onset diabetes of the young type II
NADH: nicotinamide adenine dinucleotide
NMR: nuclear magnetic resonance
PHHI: persistent hypoglycemic hyperinsulinemia of infancy
PNDM: permanent neonatal diabetes mellitus
