# Supplementary material for: Order–Disorder Transitions Govern Kinetic Cooperativity and Allostery of Monomeric Human Glucokinase
Source: PLoS Biol. 2012 Dec 18;10(12):e1001452. doi: 10.1371/journal.pbio.1001452 (PMC3525530; doi:10.1371/journal.pbio.1001452)
Supplement: Table S1 — aKinetic parameters of wild-type and variants of GCK used in this study. (DOCX) [file pbio.1001452.s010.docx]

**Table S1.** Kinetic parameters of wild-type and variant enzymes used in this study.^a^

| Enzyme | *k*_cat_ (s^-1^) | *K*_0.5, glucose_ (mM) | Hill coefficient (h) |
| --- | --- | --- | --- |
| GCK | 53 ± 1 | 8.5 ± 0.5 | 1.6 ± 0.1 |
| α13 helix variant | 41 ± 2 | 1.0 ± 0.1 | 1.2 ± 0.2 |
| GCK + 5 μM activator | 59 ± 2 | 1.3 ± 0.2 | 1.1 ± 0.1 |
| I159LI163LGCK | 15 ± 2 | 8.0 ± 0.2 | 1.2 ± 0.1 |

^a^Data represent the average of two or more independent experiments.
